# Ictal and interictal SPECT with ^99m^Tc‐HMPAO in presurgical epilepsy. I: Predictive value and methodological considerations

**DOI:** 10.1002/epi4.12786

**Published:** 2023-07-25

**Authors:** Martin Prener, Veronica Drejer, Morten Ziebell, Per Jensen, Camilla Gøbel Madsen, Svitlana Olsen, Gerda Thomsen, Lars H. Pinborg, Olaf B. Paulson

**Affiliations:** ^1^ Department of Neurology, Neurobiology Research Unit Rigshospitalet Blegdamsvej Copenhagen Denmark; ^2^ Department of Neurosurgery Rigshospitalet Copenhagen Denmark; ^3^ Department of Radiology, Centre for Functional and Diagnostic imaging and Research Copenhagen University Hospital Amager and Hvidovre Hvidovre Denmark; ^4^ Department of Neurology, Epilepsy Clinic Rigshospitalet Copenhagen Denmark; ^5^ Faculty of Health and Medical Sciences University of Copenhagen Copenhagen Denmark; ^6^ Department of Clinical Medicine University of Copenhagen Copenhagen Denmark

**Keywords:** cerebral blood flow, epilepsy surgery, neuroimaging, single photon emission computed tomography

## Abstract

**Objective:**

This retrospective study investigates the predictive value of ictal subtraction single‐photon emission computed tomography (SPECT) co‐registered to magnetic resonance imaging (MRI) (SISCOM) for successful epilepsy surgery.

**Methods:**

57 patients examined with SISCOM as a part of epilepsy surgery evaluation were divided into two groups based on seizure duration after tracer injection (group 1: Seizure duration above or equal to 30 s, group 2: Seizure duration under 30 s). SISCOM was compared to the surgical site and categorized as good or poor concordance. Subsequently, Odds ratios (ORs) and positive predictive values (PPVs) were calculated for each group for good surgical outcome, freedom from disabling seizures.

**Results:**

The PPVs and ORs for good surgical outcome was 74.1% and 5.71 for group 1 and 40% and 0.22 for group 2. SISCOM had a similar positive predictive value regardless of whether the focus was in the same or neighboring lobe, but same hemisphere as the resection.

**Conclusion:**

In conclusion, the implementation of a precise definition for a well‐executed ictal SPECT scan with respect to seizure duration after injection enhances the positive predictive value (PPV) and odds ratio (OR) for successful surgical outcome, surpassing previous findings, whether the focus in resected lobe or the neighboring.

**Key Points**

- Seizure duration after IV tracer injection should be above 30 s to ensure tracer arrival to the brain during ongoing seizures.
- Positive predictive value of scans for successful surgical outcome was 74.1% when seizure duration after tracer injection was >30 s.
- The PPV of SISCOM did not differ between the same lobe and the neighboring lobe, but the same hemisphere.

## 1 INTRODUCTION

SPECT with ^99m^Tc‐hexamethylpropyleneamine‐oxime (^99m^Tc‐HMPAO) can aid in localizing the epileptogenic focus as epileptic activity is associated with a significant increase in blood flow in affected cortical areas during epileptic activity.^1^ ^99m^Tc‐HMPAO is a special radioactive tracer that is retained in brain areas proportional to the regional cerebral blood flow (rCBF) (see methods) and ictal SPECT of ^99m^Tc‐HMPAO has been used in several epilepsy studies.^2^, ^3^, ^4^, ^5^ Subtraction Ictal by interictal SPECT and co‐registered to MRI (SISCOM) is a technique to visualize the relative increase in cerebral blood flow induced by the epileptic seizures.^6^

Considering that flow changes during seizures normalize within 2 min after the end of the seizure^1^ and that seizure activity was brief in some subjects, some measurements could have in the postictal phase. To investigate this, we divided the patients in two groups: group 1 with seizure duration after tracer injection >30 s and group 2 with a seizure duration after tracer injection of <30 s, which is the time it takes for the IV injected tracer to reach the brain.^7^

This study aimed to retrospectively evaluate the effectiveness of SPECT, for each of the above defined groups, utilizing ^99m^TC‐HMPAO in presurgical assessments of patients undergoing epilepsy surgery. The presurgical SPECT scan analysis was performed using the SISCOM method, which identifies areas with a relative increase in ictal blood flow. For our purpose, we used the original SISCOM descriptions and the surgical outcome at one‐ and two‐years follow‐up. We calculated the sensitivity, specificity, positive predictive value (PPV), and negative predictive value (NPV) of SISCOM to assess the probability of successful epilepsy surgery, freedom from disabling seizures. In a subsequent report we reanalyzed the data with especial regard to adding areas with relatively reduced flow in the SISCOM maps in addition to the traditionally mapped areas with relatively increased flow.^8^

## 2 METHODS

### 2.1 Usage of SISCOM in presurgical epilepsy evaluation—Patient inclusion

The present study is a retrospective study of the use of SISCOM in presurgical assessment in the years 2010 and 2019. In these years, 164 drug‐resistant epilepsy patients underwent Ictal and interictal SPECT with ^99m^Tc‐HMPAO during presurgical evaluation at Copenhagen University Hospital and the Epilepsy Hospital Filadelfia situated 100 km west of Copenhagen. Of these 164 patients, 58 underwent epilepsy surgery at the Department of Neurosurgery at Rigshospitalet.

The clinical indication for the scan in each individual patient was determined by the attending epilepsy physician and/or a multidisciplinary clinical team, who were ultimately responsible for making the decisions regarding epilepsy surgery.

SISCOM was primarily used in cases wherein identification of the epileptogenic focus was challenging. As a general rule of thumb, the patient's usual seizure must be above 30 s in duration. Our database for the latter part of our patient inclusion, the years 2015–2019 allows us to evaluate how frequently we used SPECT/SISCOM with ^99m^Tc‐HMPAO among the patients undergoing epilepsy surgery. Over the course of these years a total number of 165 patients underwent epilepsy surgery. Of these 30 were investigated with SPECT, representing approximately 18 percent.

### 2.2 Patient subgroups

The patients were categorized into two groups based on the duration of their seizures following the injection of the tracer. Group 1 consisting of 39 patients with seizure duration of more than or equal to 30 s after tracer injection. Group 2 consisting of the 18 patients with seizure duration of less than 30 s following tracer injection. We consider group 1 to represents a group suitable for assessment of the use of SPECT/SISCOM in presurgical evaluation of patients with epilepsy, whereas group 2 was considered to be more doubtful as they may be in a transition phase from ictal to postictal phase or in the postictal phase.

**FIGURE 1:**
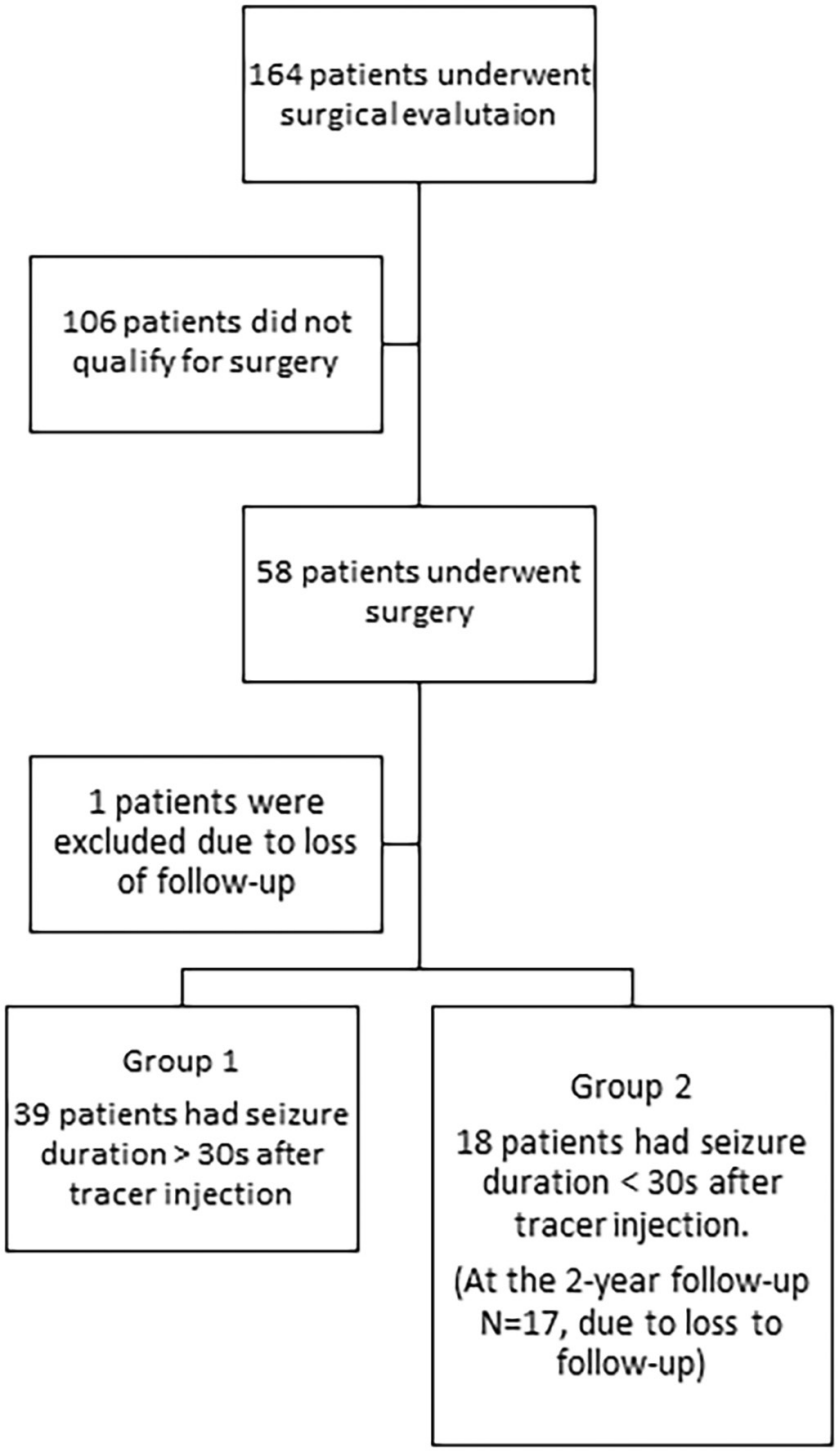
Shows a flow‐chart of patient inclusion.

One patient was excluded due to missing follow‐up, another patient in group 2 had only 1‐year follow‐up and the remaining patients had follow‐up at 1 and 2 years after surgery. Figure 1 shows an overview of patient inclusion. Evaluation of the postoperative result and methodological evaluation, seizure duration after injection, was done retrospectively. The study was approved by Danish Health and Medicines Authority (2019, record number: 3‐3013‐1030/1).

### 2.3 MR and SPECT scanning

The MRI scanner used for the co‐registration to the SPECT image was a 3‐Tesla Siemens Verio scanner, a few patients were scanned on a 3‐Tesla Trio scanner, both situated at Hvidovre Hospital. T1 weighted MPRAGE sequence (1.0 mm iso) was used.

SPECT scanning were performed using a three‐headed IRIX SPECT scanner (Philips Medical Systems) with high‐resolution parallel‐hole collimators (LEUHR‐PAR). The imaging energy window with a width of 20% was positioned at 140 keV, i.e. 126–154 keV.

Images were acquired at 120 angles with an interval of 3 degrees using noncircular orbit in a step and shoot mode. A 128 × 128 matrix with 2.33 mm isotropic voxels was used for all acquisitions. The total scanning time for each scan was 27 min (40 s pr. step in 40 steps). Images were reconstructed using an ordered subset iterative algorithm with a low pass fourth‐order Butterworth filter at 0.3 Nyquist (0.064 mm‐1) and Chang's algorithm was applied for attenuation correction with a uniform attenuation coefficient of 0.13 cm‐1. The system resolution with the LEUHR‐PAR collimators was 6.0 mm full width half maximum at a 10 cm distance from the collimator.^9^

### 2.4 The SPECT tracer ^99m^Tc‐HMPAO for cerebral blood flow measurement

^99m^Tc‐HMPAO is a lipophilic tracer that has a high permeability across the blood–brain barrier (BBB). Once it transverses the BBB it is rapidly converted to a hydrophilic compound that cannot cross back, resulting in its accumulation in the brain. The distribution of the tracer in the brain is therefore directly proportional to the rCBF at the time of tracer arrival. The majority of the tracer uptake occurs during its first pass through the brain. As the tracer is retained in the brain and sufficiently stable the SPECT scan can be postponed a few hours after tracer injection,^10^ the recommended scan being time is 30–60 min after injection, to which we complied.^9^, ^11^

A protocol was made for dosage volume in relation to time of injection, planned dosage 900 Mbq. For all groups, ictal and interictal the median tracer dosage was around 917 MBq ^99m^Tc‐HMPAO with an interquartile range in the order 887–954 MBq ^99m^Tc‐HMPAO. Minor variations in dosage between different groups are listed in Table 1.

**TABLE 1:** Patient, clinical and basic SPECT data.

	Group 1 (>30 s)	Group 2 (<30 s)
No. patients	39	18
Age at SPECT in years	29 (6–57)	19.5 (5–49)
Sex female (%)	21 (54)	9 (50)
Median seizure frequency before SPECT, no. of seizures/month	3.5 (1.5–150)	3.5 (2–240)
No. of patients with history of previous brain surgery (%)	8 (21)	0 (0)
No. of patients with one or more MRI lesions suspected to be epileptogenic, (%)	23 (59)	8 (44)
Duration of seizure after tracer injection in seconds^a^	76 (30–276)	13 (0–28)
Total seizure duration in seconds	97 (46–300)	35 (15–195)
Injection latency, time from seizure onset to injection in seconds^a^	20 (9–40)	17.5 (8–195)^b^
Injected tracer dose Ictal, MBq	925 (211–1075)	892 (160–999)
Injected tracer dose Interictal, MBq	921 (244–1088)	900 (178–1043)
Seizure types (may have more than one seizure type)		
Generalized‐tonic–clonic [GTCs] (%)	25 (64)	7 (39)
Focal aware seizure [FAS] (%)	19 (49)	10 (56)
Focal impaired awareness seizure [FIAS] (%)	33 (85)	12 (67)
Surgical focus		
Temporal (%)	26 (66)	7 (38)
Frontal (%)	6 (15)	6 (33)
Parietal (%)	1 (3)	1 (6)
Occipital (%)	2 (5)	1 (6)
Insula (%)	1 (3)	1 (6)
Other (%)^c^	3 (8)	2 (11)
Pathohistology		
Focal cortical dysplasia type IIA (%)	6 (14)	7 (38)
Ganglioglioma WHO grade I (%)	3 (8)	
Hippocampal sclerosis (%)	3 (8)	1 (6)
Malformation of cortical development (%)	2 (5)	
Dysembryoplastic neuroepithelial tumor (%)	2 (5)	1 (6)
Polymicrogyria (%)	1 (3)	
Astrocytoma WHO grade I (%)		1 (6)
Mesial temporal sclerosis (%)		1 (6)
Focal cortical dysplasia type IIB (%)	1 (3)	1 (6)
Focal cortical dysplasia type I (%)	1 (3)	
Oligodendroglial hyperplasia with hippocampal sclerosis (%)	1 (3)	
Periventricular heterotopia (%)	1 (3)	
Normal tissue (%)	15 (37)	6 (32)
Not available (%)	3 (8)	

^a^ Seizure onset: based on EEG or clinical symptoms, what comes first; Seizure end: based on clinical symptoms.

^b^ The second latest injections latency was 46 s.

^c^ Deep structures, gyrus cingula, frontotempoparietal.

*Note*: Basic patient data, clinical and SPECT data for the 39 patients included in the predictive evaluation done before surgery and the 37 patients included in the later correlative analyses done after surgery. Continuous variables are shown as median and range in parenthesis.

### 2.5 Setup and procedures

For the ictal SPECT scan the patient's antiseizure medicine was tapered off. A specialized technologist observed the patient and recorded the seizure onset time, defined by either the beginning of rhythmic ictal EEG discharges or earliest onset of clinical symptoms. At the onset of the seizure the technologist immediately injected the radiotracer, ^99m^Tc‐HMPAO, intravenously. The end of the seizure was determined solely on clinical observation. The technologist timed the entire seizure and noted the time of injection, seizure duration, injection latency and seizure duration after tracer injection, Table 1. Early injection of the radiotracer is important for an accurate interpretation of ictal SPECT data.^12^, ^13^, ^14^, ^15^

The inter‐ictal SPECT scan followed the same procedure, except the scan was conducted on an outpatient basis. To be eligible for the study, the patients had to be seizure‐free for the last 24 h prior to the scan. The interictal study was conducted in a dimply lit room, and the patients were instructed to keep their eyes open at the time of the ^99m^Tc‐HMPAO injection. The patients rested for 5 min before and after the injection.

### 2.6 SISCOM processing and analysis

SISCOM analysis was performed using the Analyze software (*Biomedical Imaging Resource, Mayo Foundation*) by subtracting the interictal SPECT from the Ictal SPECT and consequently visualizing areas of hyperperfusion with a z‐score higher than 2, i.e., the 2.4% with the relatively highest values.^12^, ^16^

This analysis of the SISCOM images was carried out by MZ and PJ.

### 2.7 Predictive evaluation

The presurgical anatomical SISCOM description was subjected to a comparison with the area of resection and a concordance score on a scale from 2 to −1 was assigned: 2 = SISCOM hyperperfusion in the same hemisphere and lobe. 1 = SISCOM hyperperfusion in same hemisphere and neighboring lobe or there are two foci, one being in the correct hemisphere and lobe and one focus in the opposite hemisphere. 0 = SISCOM showed no foci. −1 = SISCOM hyperperfusion in opposite hemisphere or hyperperfusion in same hemisphere but not neighboring lobes e.g., occipital lobe with surgical focus in the frontal lobe. The concordance scores were assigned by OP and MP.

We used the Engel classification I–IV to describe the surgical outcome at the one and 2 years follow up. Engel I = free of disabling seizures. Engel II = rare disabling seizure. Engel III = worthwhile improvement. Engel IV = no worthwhile improvement. The Engel score was obtained from the patients' medical records. We defined successful surgery outcome as Engel I and a concordance score of 1 or above was considered good, Table 2.

**TABLE 2:** 2‐year follow‐up: Predictive evaluation of SISCOM analyses performed immediately after the study and used in in the final multidisciplinary evaluation upon which decision for surgery is based.

2‐year follow‐up	Group 1 (39)				Group 2 (17)			
	Seizure duration after injection >30 s				Seizure duration after injection <30 s			
	N patients pr. Engel class				N patients pr. Engel class			
(A)	Engel I (24)	Engel II (9)	Engel III (1)	Engel IV (5)	Engel I (10)	Engel II (2)	Engel III (2)	Engel IV (3)
2	13	2	0	2	3	0	2	1
1	7	3	0	0	2	0	0	1
0	2	1	1	1	3	1	0	1
‐1	2	3	0	2	2	1	0	0
(B)	Engel I (24)		Engel II, III, IV (15)		Engel I (10)		Engel II, III, IV (7)	
2	13		4		3		3	
1, 0 and − 1	11		11		7		4	
Sensitivity	54.2				30.0			
Specificity	73.3				57.1			
PPV	76.5				50.0			
NPV	50.0				36.4			
Odds ratio	3.25 (CI_95%_: 0.80–13.15)				0.57 (CI_95%_: 0.08–4.30)			
Fisher's exact test	*P* = 0.112				*P* = 0.644			
(C)	Engel I (24)		Engel II, III, IV (15)		Engel I (10)		Engel II, III, IV (7)	
2 and 1	20		7		5		4	
0 and − 1	4		8		5		3	
Sensitivity	83.3				50.0			
Specificity	53.3				42.9			
PPV	74.1				55.6			
NPV	66.7				37.5			
Odds ratio	5.71 (CI_95%_: 1.30–25.03)				0.75 (CI_95%_: 0.11–5.24)			
Fisher's exact test	*P* = 0.031*				*P* = 1			

*Note*: Shows data for the postoperative evaluation at the 2‐year follow‐up. (A): In group 1 39 patients had been giving their Engel score and in group 2 that number was 17. B, C, and D: the data is clustered together to calculate sensitivity, specificity, PPV and NPV in %. In parentheses are the number of patients shown. The Odds ratio is further calculated with the 95% confidence interval. Last, Fisher's exact test, is used to calculate the *P*‐Value with an alpha level of 0.05.

* Statistically significant.

Using these criteria, we calculated the sensitivity and specificity, odds ratio with 95% confidence interval, and a *P*‐Value using Fisher exact test. The odds ratio (OR) is defined as ([good concordance and Engel 1]/[good concordance and Engel II‐IV])/([bad concordance and Engel 1]/[bad concordance and Engel II‐IV]). Lastly, we compared the ratio of odd ratios between group 1 and 2 for the 1‐year and 2‐year follow‐up to 1 using the Zelen test.

## 3 RESULTS

### 3.1 Clinical data

The mean age of patient in group 1 (>30 s seizure duration after IV tracer injection) and group 2 (< 30 s seizure duration after IV tracer injection) was 29 years (6–57) and 19.5 years (5–49) respectively. The median seizure frequency before SPECT was 3.5 (1.5–150) and 3.5 (2–240) seizures per month respectively for the two groups. Previous brain surgery for epilepsy had been performed in 8 (21%) in group 1, none in group 2. Furter demographic data are given in Table 1 including seizure types, surgical focus and pathohistology.

### 3.2 Latency in the SPECT studies

The median injection latency was 20 s (9–40 s) and 17,5 s (8–195 s) for group 1 and 2 respectively. In group 2 the second longest latency was 46 s (Table 1).

The median seizure duration after tracer injection for group 1 was 76 s (30–276 s) and 13 (0–28 s) for group 2 (Table 1). The median total seizure duration for group 1 was 97 s (46–300 s) and for group 2 it was 35 s (15–195 s).

**FIGURE 2:**
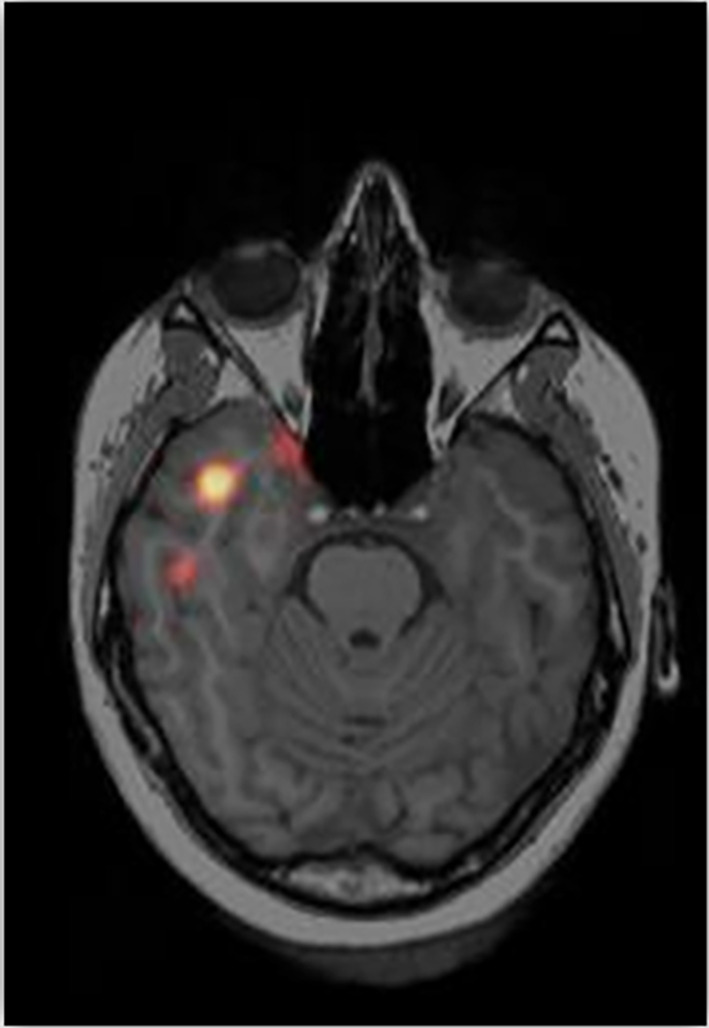
Show a clear hyperperfusion (red) focus in the anterior temporal lobe in the right hemisphere. The patient had a seizure duration after tracer injection of 45 s and an injection latency of 18 s. The outcome of surgery was Engel I.

A typical example of an optimal study with a clear epileptic focus and with good surgical outcome is shown in Figure 2.

### 3.3 Predictive values

Table 2 shows the data at the 2‐year follow‐up and Table S1 (supplementary material) shows the data at the 1‐year follow‐up. Table 2A and Table S1A present the raw predictive data, while Table 2B,C and Table S1B,C present the clustered data used for calculating sensitivity, specificity, positive predictive value (PPV), negative predictive value (NPV), odds ratio with 95% confidence interval, and Fisher's exact test with an alpha level of 0.05. In Table 2B,C and Table S1B,C Engel I is defined as good outcome, but they differ in their definition of a good concordance score. In Table 2B and Table S1B, only a score of 2 (best concordance) is considered as a good concordance score. On the other hand, in Table 2C and Table S1C, both scores of 2 and 1 are considered as good concordance.

At the 2‐year follow‐up shown in Table 2B the sensitivity for group 1 is 54% and for group 2 it is 30, while the specificity is approximately 73% and 57% respectively. In contrast, Table 2C shows that the sensitivity increases to 83% for group 1, but the specificity decreases to 50%. For group 2 the sensitivity and specificity are 50% and 43% respectively. The PPV and NPV remain unaltered at around 75% and 55% respectively in Table 2B compared to Table 2C for group 1. For group 2 PPV and NPV also remain unaltered at approximately 50% and 36% respectively. The OR for group 1 Table 2B was 3.25 and in Table 2C it was 5.71, which also was significant (*P* = 0.031). For group 2 the OR was 0.57 in Table 2B and 0.75 in Table 2C.

In Table S1 the same calculations were performed as described above at the 1‐year follow‐up. For group 1 the PPV and the NPV remained approximately the same in both Table S1B,C at 73% and 55%. For group 2 the PPV and the NPV also remained approximately the same at 42% and 30% respectively. For group 1 the OR in Table S1B was 1.66 and in Table S1C it was 2.38. For group 2 the OR was 0.43 in Table S1B and 0.22 in Table S1C.

The ratio of odd ratios (2.38 and 0.22, respectively) comparing group 1 and group 2 at the 1‐year follow‐up using Zelens test (Table 2) was 0.0936 (*P* = 0.096). At the 2‐year follow‐up the ratio of odd ratios (5.71 and 0.75, respectively) was 0.1313 (*P* = 0.21). Meaning there was not a significant difference between the OR between group 1 and group 2.

Both groups combined: Engel score where moderately consistent from the 1‐year follow‐up to the 2‐years follow‐up. 81.6% of the patient had the same Engel score at both follow‐up points. 5.3% had a lower score at the 2‐year follow‐up than at the 1‐year follow‐up. 10.5% had an Engel score 1 higher and 2.6% had an Engel score 2 higher at the 2‐year follow‐up.

### 3.4 Additional analysis

We further stratified the 57 patients into groups based on the total seizure duration. The cut‐offs were 80 s, 60 s, 45 s, as presented in Table 3 as group A and B, C and D, E and F, respectively. For the groups with long seizure duration (A, C and E) the odds‐ratio fell from 3.25 to 2.36 and to 1.48 when the cut‐off time were decreased from 80 s to 60 s to 45 s respectively.

**TABLE 3:** Total seizure duration.

	Seizure duration 80 s	
	Group A (>80 s) N = 27	Group B (<80 s) N = 30
Average seizure duration	98.8	26.5
Median seizure duration	85	26
No. of patients with seizure duration after injection under 30 s	1 (3.7%)	17 (56.7%)
Odds ratio	3.25 (0.611–17.28)	0.43 (0.09–2.15)
Sensitivity	72.2	58.8
Specificity	55.6	23.1
PPV	76.4	50
NPV	50	30

	Seizure duration 60 s	
	Group C (>60 s) N = 34	Group D (<60 s) N = 23
Average seizure duration	88.5	19.7
Median seizure duration	79.5	17
No. of patients with seizure duration after injection under 30 s	2 (5.9%)	16 (69.6%)
Odds ratio	2.36 (0.52–10.67)	0.375 (0.07–2.14)
Sensitivity	73.9	50
Specificity	45.5	27.3
PPV	73.9	42.9
NPV	54.5	33.3

	Seizure duration 45 s	
	Group E (>45 s) N = 44	Group F (<45 s) N = 13
Average seizure duration	75.1	12.2
Median seizure duration	73.5	13
No. of patients with seizure duration after injection under 30 s	4 (9.1%)	13 (100%)
Odds ratio	1.48 (0.40–5.43)	0.4 (0.04–3.96)
Sensitivity	69.0	50.0
Specificity	40.0	28.6
PPV	69.0	37.5
NPV	60.0	40.0

*Note*: 57 patient undergoing epilepsy surgery is divided into groups based on a cut‐off in total seizure duration. The cut‐off is 80 s, 60 s, and 45 s respectively. The average seizure duration, median seizure duration, no. of patients with a seizure duration after injection of above 30, OR, sensitivity, specificity, PPV and NPV for each of the subgroups is calculated.

The median injection latency was 20 s for group 1 with a seizure duration after injection of 30 s, meaning that the median total seizure duration was above 50 s. Comparing the main group 1 to the group C, with a total seizure duration above 60 s, revealed almost identical sensitivity, specificity, PPV, NPV, and odds ratio. Only 5.9% of patients in the group C, with a total seizure duration of more than 60 s had a seizure duration after injection of less than 30 s. In group D, with a total seizure duration of less than 60 s, 70% had a seizure duration after injection of less than 30 s.

## 4 DISCUSSION

### 4.1 General consideration on epileptic activity and the flow tracer ^99m^Tc‐HMPAO

The uptake of the tracer occurs during the first 60 s after IV tracer injection.^17^ If the seizure duration after tracer injection is brief, the tracer bolus may not have reached the brain before the seizure terminates, and the measurement may thus represent the early post‐seizure phase. To further assess this possibility, we have examined previous studies that measured the time profile of an intravenously injected bolus's arrival and passage through the brain.^7^ Following an intravenous bolus injection of a tracer in a cubital vein, the arterial concentrations of the tracer in the carotid artery starts to rise within 10–15 s and reaching peak values at 20 s. The peak value in the jugular vein appears about 6 s later with about half of these 6 s representing the circulation from the capillaries to the jugular vein. Considering that the ictal flow increase may persist for a few seconds after end of the seizure, it appears that the majority of the IV injected tracer bolus has reached the cerebral region within 30 s. Based on this information, we decided to use a seizure duration after IV tracer injection of at least 30 s as a cut off for a good and reliable SPECT study.

During the onset of a focal seizure, epileptic activity initiates in the seizure onset zone and subsequently spreads to other connected brain areas involved in the seizure. In the case of bilateral generalization, the spread occurs to both hemispheres. Since it takes time from the seizure onset until the tracer has been injected and for the tracer to reach the brain, SPECT with intravenous ^99m^Tc‐HMPAO injection will rarely be able to map exclusively the seizure onset zone but will also map seizure propagation to connected areas with flow increase. Consequently, it is not surprising that SPECT will map areas at some distance from the suspected seizure onset zone.

The postictal changes in cerebral hemodynamics remain an area of interest in epilepsy research. Brodersen et al.^1^ demonstrated that hyperperfusion induced by epileptic seizures normalizes after 2 min. Some studies using SPECT have attempted to characterize perfusion patterns after the end of the seizure.^18^, ^19^ However, the delay from bolus injection to arrival to the brain and the question of remaining electrical discharge after end of seizure makes it difficult to draw firm conclusions regarding the postictal flow evolution. The immediate profile of cerebral perfusion after the seizure remains largely unknown. Further research regarding rCBF following a seizure is warranted.

In view of the above consideration, we conducted the same statistical analysis for group 2 as for group 1. The sensitivity, specificity, and positive predictive value we obtained for group 2 were 40%, 25%, and 40%, respectively, at the 1‐year follow‐up. In contrast, the values for group 1 were 76%, 42.9%, and 70.4%, respectively. This finding strongly supports the notion that seizure durations of less than 30 s after tracer injection lead to less consistent interpretations, as the patient may no longer be in the ictal phase but rather in an early postictal phase.

For optimal results, the administration of the tracer should be carried out as soon as possible after seizure onset. Late injection, defined in the literature as above 45 s, has been shown to be associated with reduced data quality and decrease in the positive predictive value.^20^, ^21^ In our study, the average time between seizure onset and tracer injection for group 1 was 20 s, with the longest being 40 s. For group 2, the average time was 17.5 s, with the longest being 195 s. The second longest time was 46 s. These considerations regarding late tracer injection do not affect group 1, but they do to some extent affect group 2.

#### 4.1.1 Seizure duration after injection or total seizure duration?

The sensitivity, specificity, PPV, NPV and OR were almost identical for the group with a seizure duration after injection of more than 30 s (group 1) and the group with a total seizure duration of more than 60 s (group C). The median injection latency in group 1 with a seizure duration after injection of more than 30 s was 20 s. Thus, this is in accordance with the fact that the patients with the shortest seizure duration in group 1 had a duration of approximately around 60 s (Table 3). Comparing group 1 and group C we were thus not able to find any difference, but following the logic presented earlier in this paper, we believe seizure duration after injection is a more accurate approach.

### 4.2 Positive and negative predictive values (PPV, NPV) and odds ratio (OR) for good surgical outcome using SISCOM

The predictive values in our study are based on the descriptions of the SISCOM used in in the final multidisciplinary evaluation upon which decision for surgery was based. The PPV for both concordance score 2 and 1 (same lobe, neighboring lobe respectively) combined and for score 2 alone was roughly equivalent, at 70%–75%. This may suggest that the region visualized might not be the seizure onset zone, but instead, represent neighboring region with epileptic activity due to seizure propagation. Thus, SISCOM might show a neighboring area, and the seizure onset zone might have gone silent. In a meta‐analysis study conducted by Chen et al, comprising 10 studies the average PPV was 56.^5^ The lower PPV value observed in comparison to our study may, at least in part, be explained by the fact that neither did they use a lower limit for the seizure duration following tracer injection nor an upper limit for seizure duration before tracer injection. Further, they defined good concordance as a SISCOM focus lateralized to the operated hemisphere whereas our definition was slightly more rigid, as we defined good concordance as a score of at least 1, meaning neighboring region.

In group 1 the NPV was around 50%, indicating that a low SISCOM concordance score is not valuable for clinical assessment. In group 2 the NPV ranged from 25 and 37%. A previous study by Schneider et al^2^ with 14 patients found an NPV of 67%.

In our study the OR the correlation for good surgical outcome and a concordance score of 2 was 1.66 and 3.25 for the 1‐year and 2‐year follow up respectively. These findings are consistent with the overall OR of 3.28 (ranging from 0.22 to 18.33) reported in the meta‐analysis by Chen et al.^5^

At the 1‐year follow‐up, the ratio of odd ratios was 0.0936 (*P* = 0.096) and at the 2‐year follow‐up it was 0.1313 (*P* = 0.21). The estimated odd ratios in group 1 and 2 were numerically quite different, those of group 1 typically above 1 and those of group 2 below 1. However, due to the small sample size, the uncertainty surrounding these estimates was substantial, and as a result, there was insufficient evidence to suggest a group difference. Additional research is necessary to determine the presence or lack of group differences.

A recent study by Aungaroon et al 2018^22^ observed that the sensitivity, specificity, PPV, NPV and odds ratio increased with a cut‐off of 20 s seizure duration after injection. They further stratified seizure duration after injection and found that for each second the seizure duration after injection increased, the odds of a localizing SISCOM went up by 7%, although not significantly (*P* = 0.06). They used the term “localizing SISCOM” to refer to SISCOM hyperperfusion in the same areas as the later resection. As demonstrated earlier, we showed that including SISCOM with a concordance score of 1, meaning the neighboring region, did not negatively affect the PPV. Despite this difference, our findings are consistent well with theirs.

### 4.3 Sensitivity and specificity for good surgical outcome using SISCOM

The *sensitivity* for Engel I of the predictive evaluation in group 1 was 76% and 83.3% at the 1‐year and the 2‐years follow‐up, respectively, when combining SISCOM concordance score 1 and 2 (Table 2C). These findings are consistent with a meta‐analysis that reported sensitivity values for Ictal SPECT to range between 66% and 97%.^23^ Restricting the concordance score to 2 (the exact epileptogenic lobe for the SISCOM focus) decreased the sensitivity to approximately 50% both at the 1‐year and 2‐years follow‐up. This observation may indicate that the area visualized by SISCOM might not represent the seizure onset zone but rather the seizure propagation to neighboring areas, and the seizure onset zone might even have gone silent. This correspond to the PPV for the combined concordance score 2 and 1, and only 2 being roughly the same as previously discussed.

The *specificity* in the present study was between 65% and 73% when only concordance score of 2 was regarded as good concordance. When concordance score of 1 was included, the specificity decreased to approximately 50%. Most studies reporting the specificity of SISCOM/SPECT compares the focus of the modality to the presumed epileptogenic zone. Schneider et al.^2^ reported a specificity of 50% for seizure‐free outcome in a study of 14 patients, which is consistent with our findings.

## 5 LIMITATIONS

The study was conducted retrospectively, thus the surgical results were known to the authors, although the SISCOM scans were described prior to the surgery, as a part of the presurgical evaluation. Another limitation is the relatively small sample size, especially in group 2 with only 17 patients at the 2‐year follow‐up. There is a variation in seizure types between the two groups, with group 1 predominantly having temporal seizures and group 2 having almost equal frequency of frontal and temporal seizures. Lastly, there is an obvious difference in seizure duration between the two patient groups, with the shortest duration in group 2. Statistical analysis regarding histopathology and seizure onset location (lobe) were not feasible due to small sample size.

## 6 CONCLUSION

SISCOM is a valuable diagnostic tool for obtaining knowledge of the epileptogenic lesion in patients with drug resistant epilepsy. Although SISCOM does not provide a precise target for resection, it can give clinicians more confidence in their search for the epileptogenic zone. Therefore, in our study, SISCOM was primarily used in cases where the location of the epileptogenic zone was difficult to determine, such as in cases with MRI‐negative findings. Our study demonstrates a moderate sensitivity and PPV of SISCOM for freedom from disabling seizures, which is in concordance with previous literature.

The analysis of the results raises methodological considerations. For instance, the seizure duration after radiotracer injection needs to be taken into account as it takes time for the intravenously injected tracer to reach the brain. Additionally, a SISCOM focus in the neighboring lobe but same hemisphere did not affect the PPV. We therefore conclude that a more stringent definition of a correctly performed ictal SPECT scan, seizure duration after tracer injection >30 s, and including the neighboring lobe, would increase the reliability of the modality.

## AUTHOR CONTRIBUTIONS

MP: Analyzed data, drafted manuscript. VD: analyzed data. MZ: Described the SPECT scans prior to surgery. PJ: Described the SPECT scans prior to surgery. CGM: Described MRI scans. SO: Performed the ictal and interictal scans and generated the SISCOM images. GT: Performed the ictal and interictal scans and generated the SISCOM images. LP: Recruited the patients. OBP: Planed and supervised the analysis of the material. Drafted manuscript. All authors participated in drafting the final version of the manuscript.

## CONFLICT OF INTEREST STATEMENT

Neither of the authors has any conflict of interest to disclose.

## ETHICS STATEMENT

We confirm that we have read the Journal's position on issues involved in ethical publication and affirm that this report is consistent with those guidelines.

## CLINICAL TRIAL STATEMENT

The present study is a retrospective evaluation of the use of SPECT with the tracer ^99m^Tc‐HMPAO in presurgical investigation of patients with epilepsy as introduced in the clinical work in the years 2010–2019. It was introduced for clinical purposes and not registered as a clinical trial.

## Supporting information


Table S1.

## Data Availability

Data can be obtained by contacting the authors. The access to and use of the data must be in accordance with the rules of the Danish legislation and must be approved according to the Danish Data Protection Agency's rules.
