# Supplementary material for: Ictal and interictal SPECT with 99mTc‐HMPAO in presurgical epilepsy. I: Predictive value and methodological considerations
Source: Epilepsia Open. 2023 Jul 25;8(3):1064–74. doi: 10.1002/epi4.12786 (PMC10472396; doi:10.1002/epi4.12786)
Supplement: Supplementary file 1 — Table S1. [file EPI4-8-1064-s001.docx]

**Supplementary Materials**

**Table S1. 1-year follow-up: Predictive evaluation of SISCOM analyses performed immediately after the study and used in in the final multidisciplinary evaluation upon which decision for surgery is based.**

| **1 year follow-up** | Group 1 (39) Seizure duration after injection >30s | | | | Group 2 (18) Seizure duration after injection <30s | | | |
| --- | --- | --- | --- | --- | --- | --- | --- | --- |
|  | N patients pr. Engel class | | | | N patients pr. Engel class | | | |
| **A)** | Engel I (25) | Engel II (7) | Engel III (3) | Engel IV (4) | Engel I (10) | Engel II (2) | Engel III (5) | Engel IV (1) |
| **2** | 12 | 3 | 1 | 1 | 3 | 1 | 3 | 0 |
| **1** | 7 | 3 | 0 | 0 | 1 | 0 | 1 | 1 |
| **0** | 3 | 0 | 1 | 1 | 3 | 1 | 1 | 0 |
| **-1** | 3 | 1 | 1 | 2 | 3 | 0 | 0 | 0 |
| **B)** | Engel I (25) | | Engel II, III, IV (14) | | Engel I (10) | | Engel II, III, IV (8) | |
| **2** | 12 | | 5 | | 3 | | 4 | |
| **1, 0 and -1** | 13 | | 9 | | 7 | | 4 | |
| **Sensitivity** | 48.0 | | | | 30.0 | | | |
| **Specificity** | 64.3 | | | | 50.0 | | | |
| **PPV** | 70.6 | | | | 42.9 | | | |
| **NPV** | 59.1 | | | | 36.4 | | | |
| **Odds ratio** | 1.66 (CI_95%_: 0.43 – 6.38) | | | | 0.43 (CI_95%_: 0.06 – 2.97) | | | |
| **Fisher`s exact test** | *p=0.518* | | | | *p=0.631* | | | |
| **C)** | Engel I (25) | | Engel II, III, IV (14) | | Engel I (10) | | Engel II, III, IV (8) | |
| **2 and 1** | 19 | | 8 | | 4 | | 6 | |
| **0 and -1** | 6 | | 6 | | 6 | | 2 | |
| **Sensitivity** | 76.0 | | | | 40.0 | | | |
| **Specificity** | 42.9 | | | | 25.0 | | | |
| **PPV** | 70.4 | | | | 40.0 | | | |
| **NPV** | 50.0 | | | | 25.0 | | | |
| **Odds ratio** | 2.38 (CI_95%_: 0.60 - 9.64) | | | | 0.22 (CI_95%_: 0.03 – 1.71) | | | |
| **Fisher`s exact test** | *p=0.287* | | | | *p=0.188* | | | |

Table S1 shows data for the postoperative evaluation at the 1-year follow-up. A): In group 1 39 patients had been giving their Engel score and in group 2 that number was 18. B, C, and D: the data is clustered together to calculate sensitivity, specificity, PPV and NPV in %. In parentheses are the number of patients shown. The Odds ratio is further calculated with the 95% confidence interval. Last, Fisher`s exact test, is used to calculate the p-value with an alpha level of 0.05.
